# Antioxidation and symbiotic nitrogen fixation function of *prxA* gene in *Mesorhizobium huakuii*

**DOI:** 10.1002/mbo3.889

**Published:** 2019-06-09

**Authors:** Sanjiao Wang, Tiantian Lu, Qiang Xue, Ke Xu, Guojun Cheng

**Affiliations:** ^1^ College of Life Sciences South‐Central University for Nationalities Wuhan Hubei China; ^2^ Hubei Provincial Engineering and Technology Research Center for Resources and Utilization of Microbiology, College of Life Sciences South‐Central University for Nationalities Wuhan Hubei China

**Keywords:** antioxidant and symbiotic gene expression, antioxidant function, *Mesorhizobium huakuii*, peroxiredoxin gene *prxA*, symbiotic nitrogen fixation

## Abstract

Peroxiredoxins (Prxs) play an essential role in the antioxidant activity and symbiotic capacity of *Mesorhizobium huakuii*. A mutation in the *M. huakuii prxA* gene (encoding a Prx5‐like peroxiredoxin) was generated by homologous recombination. The mutation of *prxA* did not affect *M. huakuii* growth, but the strain displayed decreased antioxidative capacity under organic cumene hydroperoxide (CUOOH) conditions. The higher resistance of the* prxA* mutant strain compared with the wild‐type strain to more than 1 mmol/L H_2_O_2_ was associated with a significantly higher level of glutathione reductase activity and a significantly lower level of intracellular hydrogen peroxide content. Real‐time quantitative PCR showed that under 1 mmol/L H_2_O_2_ conditions, expression of the stress‐responsive genes *katG* and *katE* was significantly upregulated in the *prxA* mutant. Although the *prxA* mutant can form nodules, the symbiotic ability was severely impaired, which led to an abnormal nodulation phenotype coupled to a 53.25% reduction in nitrogen fixation capacity. This phenotype was linked to an absence of bacteroid differentiation and deregulation of the transcription of the symbiotic genes *nifH*, *nifD*, and *fdxN*. Expression of the *prxA* gene was induced during symbiosis. Thus, the PrxA protein is essential for antioxidant capacity and symbiotic nitrogen fixation, playing independent roles in bacterial differentiation and cellular antioxidative systems.

## Graphical abstract

The *Mesorhizobium huakuii* prxA mutant induced partially effective nodules on *Astragalus sinicus*. The prxA mutant nodules had thickened cell walls in the cortex and clearly decreased the number of bacteroids. The size and structure of the bacterial strain in the mutant nodule cells indicated a general lack of bacterial differentiation into bacteroids.

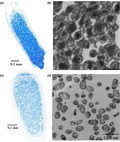

## 1 INTRODUCTION

Nitrogen is one of the most essential nutrients that are vital to plant growth. The effect of nitrogen on plant growth and development is obvious. Biological nitrogen fixation refers to the process in which nitrogen‐fixing microorganisms reduce nitrogen in the atmosphere to ammonia, which plays a very important role in agricultural production (Olivares, Bedmar, & Sanjuán, 2013). Among these nitrogen‐fixing microorganisms, the symbiotic system of rhizobia‐leguminous plants has the strongest nitrogen fixation capacity, which is of great significance and value for research (Ferguson et al., 2010). The gram‐negative soil bacteria *Rhizobium* spp. can establish an effective nitrogen‐fixing symbiosis with its legume crops through exchanging signal molecules. The symbiosis between the host plants and the rhizobia starts with the specific recognition of signal molecules produced by each partner (Bladergroen & Spaink, 1998). The plant releases a chemical signal molecule (flavonoid) that is perceived by the bacterium *Rhizobium*. In response, the Rhizobium activates transcription of *nod* genes and produces lipo‐chitooligosaccharides (Nod factors), which provoke tight root hair curls (Gressent et al., 2002). Subsequently, rhizobia enter their host cells via endocytosis and develop infection threads that are filled with proliferating bacteria. The infection threads elongate toward root hairs and penetrate the root layers. Bacteria differentiate into large pleiomorphic, nitrogen‐fixing bacteroids, leading to cell division and the consequent formation of nodules in which bacteroids reduce atmospheric nitrogen to ammonia, which is used by leguminous plants as a nitrogen source (Brewin, 2004).

During the infection process and nitrogen‐fixing symbiosis, rhizobia are greatly dependent on antioxidant systems to protect themselves against oxidant damage (Dombrecht et al., 2010). Numerous antioxidative systems can contribute to maintaining certain levels of reactive oxygen species (ROS), which include antioxidant enzymes such as catalases, glutathione (GSH), glutathione peroxidases (Gpxs), superoxide dismutases and a recently identified rapidly growing family of peroxiredoxins (Prxs) (Duan et al., 2013). Prxs are cysteine‐dependent peroxidases of the thioredoxin family that react with hydrogen peroxide, larger hydroperoxide substrates, and peroxynitrite through the formation of a cysteine sulfenic acid (R‐SOH) at the active site (Parsonage et al., 2015). An intriguing biophysical property of Prxs is the redox‐dependent modulation of their oligomeric state between decamers and dimers within the physiological concentration range (Parsonage et al., 2005). Prxs are ubiquitously distributed in all organisms, including plants and bacteria as well as animals, to protect cells against oxidative stress‐induced damage (Umate, 2010). Thus far, six isoforms of Prxs have been characterized in mammals, and all of the Prxs are implicated, via their antioxidant activity, in intracellular signaling and in the regulation of processes such as cell proliferation and differentiation and protection of other proteins from oxidative damage (Lim et al., 1998). Moreover, Prxs have also been shown to be induced by oxidative stress and to be associated with the development, progression, and drug resistance of cancer (Duan et al., 2013). In *Rhizobium etli*, the peroxiredoxin gene *prxS* is strongly expressed under microaerobic conditions and plays an important role against oxidative stress during symbiotic interactions with the host *Phaseolus vulgaris* (Dombrecht et al., 2010). In rhizobia‐*Pisum sativum* symbiosis, a reduction in the nodule cytosolic peroxiredoxin contributes to nodule senescence (Groten et al., 2006).

*Astragalus sinicus* (Chinese milk vetch) is an important winter‐growing green manure that has also been used to fertilize rice fields in China and Japan for 2,000 years.* Mesorhizobium huakuii* nodulated only on its host plant, *A. sinicus*, forming indeterminate nodules (Cheng, Li, & Zhou, 2006). *Mesorhizobium huakuii* 7653R *prxA* encodes a protein with peroxiredoxin activity. In this study, mutations in the *prxA* gene were constructed by homologous recombination, and the role of *M. huakuii prxA* in oxidative stress and during nitrogen‐fixing symbiosis was investigated by analyzing the phenotypes of the mutant strain.

## 2 MATERIALS AND METHODS

### 2.1 Bacterial strains, plasmids, and culture conditions

The strains, plasmids, and primers used in this study are listed in Table 1.* Mesorhizobium huakuii* 7653R and HKprxA (*prxA* mutant) were grown at 28°C in either Tryptone Yeast extract (TY) (Beringer & Hopwood, 1976) or acid minimal salt medium (AMS) (Poole, Blyth, Reid, & Walters, 1994) with 10 mM D‐glucose or 10 mM di‐sodium succinate as carbon sources and 10 mM NH_4_Cl as a nitrogen source. In cultures grown in AMS medium with defined potassium levels, K_2_HPO_4_ was replaced by Na_2_HPO_4_, and KCl was added to appropriate concentrations. Antibiotics were used at the following concentrations (μg/ml): streptomycin (Str), 500; kanamycin (Km), 20; neomycin (Neo), 80 or 250 (for generating the *prxA* mutant); gentamicin (Gm), 20; carbenicillin (Carb), 50; and spectinomycin (Spec), 100. To monitor culture growth,* M. huakuii* strains were grown at 28°C with shaking at 200 rpm, and the optical density at 600 nm (OD_600_) was measured during the culture period.

**Table 1:** Strains, plasmids, and primers used in this experiment

Strains	Description	Reference, Source, Sequence
7653R	Wild‐type, Nod^+^on *Astragalus sinicus*	Cheng et al. (2006)
HKprxA	Rlv3841 *prxA*:: pKprxA, Str^r^ Neo^r^	This study
HKprxA(pBBRprxA)	MKprxA carrying* prxA* gene, Str^r^ Neo^r ^Gm^r^	This study
DH5α	F^‐^ lacZDM15 recA1 hsdR17 supE44 D (lacZYA argF)	This study
		
Plasmids		
pBBR1MCS‐5	Broad‐host‐range cloning vector, Gm^r^	Kovach. , Phillips, Elzer, Roop, & Peterson, 1994
pK19mob	pK19mob pUC19 derivative lacZ mob; Km^r^	Schafer et al., 1994
pRK2013	Helper plasmid used for mobilizing plasmids; Kmr	Figurski & Helinski, 1979
pKprxA	prxAUP/prxALW PCR product in pK19mob, Km^r^	This study
pBBRprxA	prxAhbUP/prxAhbLW PCR product in pBBR1MCS‐5, Gm^r^	This study
Primer^a^		
prxAUP	Sense primer for *prxA* mutation	TTTTCTAGAGTTCGCGATGATTCAGTCCA
prxALW	Antisense prime for *prxA* mutation	TTTGGATCCAGCTCTTGACCGCGGCCAGT
prxAMP	Mapping PCR primer for* prxA*	TTCGTCACGTTTCTTACGCG
pK19A	pK19mob mapping primer	ATCAGATCTTGATCCCCTGC
pK19B	pK19mob mapping primer	GCACGAGGGAGCTTCCAGGG
prxAhbUP	Sense primer for *prxA* complementation	TTTTCTAGACCGGCTATGAGGAGTGGGTG
prxAhbLW	Antisense prime for* prxA* complementation	TTTGGTACCTGACCTTTGAATGACCTCAC
QZYY‐16SUP	Sense primer for qRT‐PCR of 16S rDNA	AACTGAGATGGCTTTTGGAG
QZYY‐16SLW	Antisense primer for qRT‐PCR of 16S rDNA	GGATGACGTCAAGTCCTCAT
QZYY_nifDUP	Sense primer for qRT‐PCR of *nifD*	AGTGCATCTGACGGAACGGT
QZYY_nifDLW	Antisense primer for qRT‐PCR of *nifD*	ACCGGTTACGAGTTGGAAAA
QZYY_nifHUP	Sense primer for qRT‐PCR of *nifH*	TTGGCGCTCGTTGCAGATCA
QZYY_nifHLW	Antisense primer for qRT‐PCR of *nifH*	TTGTCATGTCCGGCGAGATG
QZYY_HmusUP	Sense primer for qRT‐PCR of *Hmus*	CAATGTTGTTCTAACCGCTC
QZYY_HmusLW	Antisense primer for qRT‐PCR of *Hmus*	GTGAGCACCTTGTCGTAGAT
QZYY_RhtAUP	Sense primer for qRT‐PCR of *RhtA*	GGCTTTCGTGCAGGAACAGG
QZYY_RhtALW	Antisense primer for qRT‐PCR of* RhtA*	CTGGAGATGGTGGCGCTGAC
QZYY_katGUP	Sense primer for qRT‐PCR of *katG*	GAATTCAAAAGCCTCGACCT
QZYY_katGLW	Antisense primer for qRT‐PCR of *katG*	GATGAACAGCGGGCCGTAAT
QZYY_katEUP	Sense primer for qRT‐PCR of *katE*	GGTATTCTTCATTCAGGATG
QZYY_katELW	Antisense primer for qRT‐PCR of *katE*	ATCCCAGAAATTGTCGTGCG
Qhkprx_for	Sense primer for qRT‐PCR of *prxA*	CCCAGTTTCGAAGACCTCTA
Qhkprx_rev	Antisense primer for qRT‐PCR of *prxA*	TCGACTTTCTGCAGCCCCAA

a Restriction sites in primer sequences are underlined.

### 2.2 Construction and complementation of the *prxA* gene mutant of *M. huakuii* 7653R

Primers MKprxUP and MKprxLW were used to PCR amplify the *prxA* region from *M. huakuii* 7653R genomic DNA. The 640‐bp *prxA* PCR product was cloned into the *Bam*HI and *Xba*I sites of pK19mob‐producing plasmid pKprxA. The plasmid pKprxA was conjugated with *M. huakuii* strain 7653R using pRK2013 as a helper plasmid as previously described (Poole et al., 1994). Insertions into the *prxA* gene of strain 7653R were confirmed by PCR using prxAMP and a pK19mob‐specific primer (either pK19A or pK19B) (Karunakaran et al., 2010).

To complement the *prxA* mutant, primers prxAhbUP and prxAhbLW were used to amplify the complete *prxA* gene from 7653R. The PCR product was digested with *Bam*HI and *Xba*I and cloned into pBBR1MCS‐5, resulting in plasmid pBBRprxA. Plasmid pBBRprxA was conjugated into the mutant strain HKprxA using pRK2013 as a helper plasmid to provide the transfer genes as previously described (Karunakaran et al., 2010).

### 2.3 Antioxidation experiments

The logarithmic phase (OD_600_: 0.3–0.6) mutant strain HKprxA and wild‐type 7653R were collected and washed twice in sterile phosphate‐buffered saline (PBS) (1X; 136 mM NaCl, 2.6 mM KCl, 8.0 mM Na_2_HPO_4_, 1.5 mM KH_2_PO_4_). Cells were treated with H_2_O_2_ and CUOOH at different concentrations (0, 0.5 mmol/L,   mmol/L, 5 mmol/L) for 1 hr. Strains were thoroughly washed with distilled water to remove any remaining oxidants, and the diluted TY plate method was used to evaluate the bacterial survival rate. The experiment consisted of three repetitions for each treatment.

### 2.4 Enzyme activity experiments

For analysis of enzymatic and nonenzymatic antioxidant activities, PBS cells were collected by centrifugation at 5,000 rpm for 5 min at 4°C. The cells were held in an ice‐water bath and sonicated for 15 min. The sonicate was centrifuged at 12,000 rpm for 10 min at 4°C. Glutathione reductase activity was determined according to the method of Di Ilio, Polidoro, Arduini, Muccini, & Federici, 1983. GSH content was determined according to the method of Irfan, Aruna, and Saibal (2006). Hydrogen peroxide content was measured as previously described (Maisonneuve, Fraysse, Lignon, Capron, & Dukan, 2008), and peroxidase activity was determined using a peroxidase assay kit (Beyotime, China).

### 2.5 Plant growth and acetylene reduction assays

*Astragalus sinicus* was used as a host plant to test nodulation of the *M. huakuii* strains. Seeds were surface‐sterilized for 5 min in 75% ethanol, soaked 20 min in 2% sodium hypochlorite, and then rinsed 10 times with sterile water. Inoculation with *M. huakuii* was performed on 7‐day‐old seedlings. Plants were incubated in a controlled‐environment chamber with an 18‐hr photoperiod (day/night temperature, 22°C and 20°C) and were harvested at 4 weeks postinoculation (Poole et al., 1994). The acetylene reduction in the plants was detected by gas chromatographic measurement as previously described (Allaway et al., 2010).

### 2.6 RNA isolation and quantitative RT‐PCR analysis

To determine the gene expression level, total RNA was isolated with Trizol reagent from free‐living *M. huakuii* 7653R cultured in AMS liquid medium, free‐living cells treated with 1 mmol/l H_2_O_2_ for 1 hr, or plant nodules harvested from *A. sinicus* inoculated with wild‐type strain 7653R or the mutant HKprxA at 2, 4, 6 weeks postinoculation. cDNA was synthesized using SuperScript^™^ II RT and random hexamers (Invitrogen). Quantitative real‐time PCR was carried out using SYBR Premix ExTaq (Takara, Dalian, China) on the Bio‐Rad CFX96 Real‐Time PCR Detection System. Primers for *prxA*, *katG*, *katE*, *fdxN*, *humS*, *rhtA*, *nifD*, and* nifH* are detailed in Table 1 (Mulley et al., 2011). The 16S rRNA gene was used as a calibrator gene, and the results were analyzed as previously described (Prell, Bourdès, Karunakaran, Lopez‐Gomez, & Poole, 2009).

## 3 RESULTS

### 3.1 Bioinformation analysis of the *M. huakuii PrxA* gene

*Mesorhizobium huakuii* MCHK_7135, encoding the peroxiredoxin PrxA, is expressed at high levels during symbiosis. The PrxA gene is predicted to encode a 177‐amino acid polypeptide with an expected molecular mass of 19.9 kDa and a pI value of 4.87. A BLASTP search (BLOSUM62 similarity matrix) against the GenBank databases showed that PrxA has a strong overall amino acid conservation (63%–97%, identity) to the peroxiredoxins from *Mesorhizobium* spp. The *M. huakuii* PrxA protein has three conserved cysteine residues namely: C56, C81, and C156. The conserved domain (RPS‐BLAST) revealed that PrxA showed homology to the PRX5‐like subfamily, which includes a cysteine‐dependent homodimeric TRX peroxidase. Although members of the PRX5‐like family have intensive ROS scavenging activity and have been widely researched in human tissues (Park et al., 2016), there are still many uncertainties about their functions in bacteria, particularly in *Rhizobium*.

### 3.2 Construction and antioxidation analysis of the *M. huakuii prxA* mutant

To confirm the function of the *prxA* gene in antioxidation and symbiotic ability, a single‐crossover integration mutation in *prxA* was constructed by the homologous recombination method. In liquid TY or AMS minimal medium with succinate or glucose as a carbon source, there is no significant difference in growth between the *prxA* mutant and wild‐type 7653R (data not shown). The importance of PrxA for protection against oxidative stress was investigated by carrying out survival assays to determine the resistance of the mutant HKprxA to inorganic oxide hydrogen peroxide (H_2_O_2_) and organic cumene hydroperoxide (CUOOH). The survival rates of HKprxA were not significantly affected by H_2_O_2_ treatments at low concentrations of 0.1 and 0.5 mmol/L compared with the wild‐type 7653R strain, whereas the antioxidative capacity of HKprxA was significantly promoted by these treatments with H_2_O_2 _at higher concentrations of 1 and 5 mmol/L (Table 2). Organic oxidative stress induced by CUOOH was also studied, as shown in Table 2. The *prxA* mutant inhibited antioxidative capacity under CUOOH oxidative stress conditions. Thus, PrxA may play different roles in oxidative stresses in *M. huakuii*.

**Table 2:** Tolerance of stains to different concentrations of H_2_O_2 _and CUOOH

Oxidant	Strain *M. huakuii*	concentration CFU/mL			
		c(oxidant)/(mmol/L)			
		0	0.5	1	5
H_2_O_2_	7653R	(1.3 ± 0.1) × 10^9^	(8.5 ± 1.2) × 10^8^	(6.9 ± 1.3) × 10^8^	(5.8 ± 0.6) × 10^8^
	MHprxA	(1.3 ± 0.1) × 10^9^	(1.1 ± 0.18) × 10^9^	(1.65 ± 0.09) × 10^9^ a	(1.0 ± 0.1) × 10^9^ a
CUOOH	7653R	(1.0 ± 0.6) × 10^8^	(7.9 ± 1.2) × 10^7^	(9.2 ± 3.3) × 10^6^	(6.8 ± 1.4) × 10^3^
	MHprxA	(1.0 ± 0.5) × 10^8^	(1.2 ± 0.7) × 10^7^ a	(1.3 ± 0.3) × 10^6^ a	(3.3 ± 0.3) × 10^2^ a

Abbreviation: CUOOH, capacity under organic cumene hydroperoxide.

a Shows significant difference compared to *prxA* mutants and 7653R at *p* ≤ 0.05.

### 3.3 Enzyme activity experiments

Protein S‐glutathionylation is a reversible posttranslational modification with critical roles in oxidative stress and signal transduction, and peroxiredoxin is involved in the regulation of intracellular glutathione redox balance (Dulce, Schulman, & Hare, 2011). The role of *M. huakuii* PrxA in controlling protein glutathionylation status was investigated by quantifying peroxidase and glutathione reductase activity and hydrogen peroxide and GSH content in 1 mM H_2_O_2_‐induced oxidative stress conditions. The results showed that the peroxidase activity and GSH content of mutant HKprxA were not different from that of wild‐type strain 7653R, but its hydrogen peroxide content was significantly lower (Table 3). The glutathione reductase activity of mutant HKprxA was higher than that of wild‐type strain 7653R and displayed a significant difference between mutant HKprxA and wild‐type 7653R (Table 3). These results indicated that the *prxA* gene played an important role in the glutathione redox balance of rhizobia.

**Table 3:** The enzymatic and nonenzymatic antioxidant activities of *Mesorhizobium huakuii* strains*

Strains *M. huakuii*	Peroxidase activity (U/mg)	Glutathione reductase activity (U/mg)	Hydrogen peroxide content (UM)	GSH (UM)
7653R	1.29 ± 0.215^a,b^	0.4 ± 0.008^a,b^	3.67 ± 0.018^a,b^	0.094 ± 0.016^a,b^
HKprxA	1.16 ± 0.274^a,b^	0.51 ± 0.032^b^	3.27 ± 0.117^b^	0.094 ± 0.017^a,b^

Note

*The data are the average of five replicates.

^a,b^ Values in each column followed by the same letter are not significantly different (*p* ≤ 0.05).

### 3.4 Plant properties of the *prxA* mutant and wild‐type strain

To assess the nodulation and nitrogen‐fixing capacity of the *prxA* mutant strain, *A. sinicus* seedlings were inoculated with the *prxA* mutant HKprxA or wild‐type 7653R, and 28 days later, nodule numbers per plant and acetylene reduction activity were measured. No significant difference in the number of nodules was observed between plants inoculated with the *prxA* mutant strain and plants inoculated with the wild‐type 7653R strain (Table 4). A highly significant feature of our study was that the *prxA* mutant induced partially effective nodules on *A. sinicus*. The *prxA* mutant elicited more spherical, rather than elongated, nodules compared to the wild‐type and showed a 53.25% decrease in acetylene reduction activity compared to the wild‐type (Table 4). When *prxA* on an environmentally stable plasmid (pBBR1MCS‐5) was introduced into mutant HKprxA, plants inoculated with the resulting strain HKprxA(pBBRprxA) formed normal nodules and showed nitrogen‐fixing ability at the same rate as did 7653R‐inoculated plants (Table 4).

**Table 4:** Symbiotic phenotypes of *Mesorhizobium huakuii* strains

Strain *M. huakuii*	Number of total nodules per plant*	Acetylene reduction activity (nmol of ethylene/plant/h)	Acetylene reduction activity (nmol of ethylene/nodulet/h)
7653R	17 ± 5.29^a,b^	33.58 ± 2.02^a,b^	2.14 ± 0.80^a,b^
HKprxA	15.3 ± 1.53^a,b^	15.70 ± 0.72^a,b^	1.03 ± 0.10^a,b^
HKprxA(pBBRprxA)	17.7 ± 4.43^a,b^	30.22 ± 2.21^a,b^	1.79 ± 0.43^a,b^
Control^n^	0	0	0

Note

*Data are the average of at least three replicates. Acetylene reduction activity of nodules induced by *prxA* mutant strain HKprxA or complementary strain HKprxA(pBBRprxA) was compared to that of nodule induced by the wild‐type strain 7653R.

Control: plants not inoculated with rhizobia strain.

^a,b^ Values in each column followed by the same letter are not significantly different (*p* ≤ 0.05).

### 3.5 RNA isolation and quantitative RT‐PCR analysis

**Figure 1:**
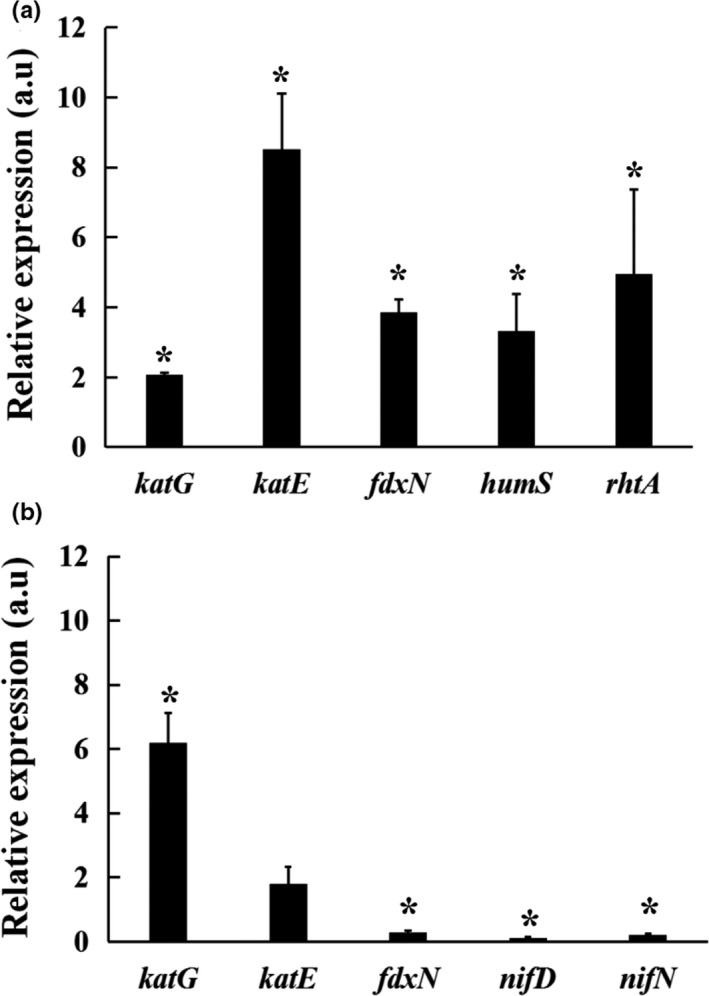
Relative expression of genes involved in *prxA* mutant in hydrogen peroxide stresses (a) and 4‐week‐nodule bacteroids (b) in* prxA* mutant compared with wild‐type 7653R measured by qRT‐PCR. Data are the average from three independent biological samples (each with three technical replicates). Statistical analysis of data sets was performed using REST (Pfaffl, Horgan, & Dempfle, 2002). Superscript asterisk indicates significant difference in relative expression (>2‐fold, *p* ≤ 0.05)

Cellular peroxiredoxins are required for protection against oxidative stress. The expression of *katG* and *katE* genes is mainly regulated at the transcriptional level in oxidative stress conditions. As shown in Figure 1a, under 1 mmol/L H_2_O_2_‐induced oxidative stress conditions, the expression of *katG* and *katE* was significantly upregulated in the *prxA* mutant, suggesting that the *prxA* mutant strain might significantly modify the cellular redox state and increase catalase gene expression.

It has been reported that Prxs which are involved in iron homeostasis and iron‐regulated pathways are connected to oxidative stress resistance (Blaiseau, Lesuisse, & Camadro, 2001; Matte et al., 2018). The possible involvement of *prxA* in iron homeostasis was investigated by assessing the ability of* prxA* deficiency to activate the expression of iron‐regulated genes. In rhizobia, two major transcription factors, Iron Responsive Regulator (*irr* gene) and *Rhizobium* Iron Responsive A (rirA), are involved in the regulation of iron homeostasis. Genes *rhtA* and *hmuS*, which encode proteins involved in iron transport, were regulated by RirA (Benyamina, Baldacci‐Cresp, Couturier, Chibani, & Frendo, 2012). The messenger RNA expression of *rhtA* and *hmuS* was studied using reverse transcription polymerase chain reaction in wild‐type 7653R and *prxA* mutant strains. Genes *rhtA* and *hmuS* were significantly overexpressed in *prxA* compared with wild‐type 7653R. Thus, *prxA* is involved in the regulation of iron homeostasis through the modulation of RirA. Since a large reduction in the nitrogen‐fixing capacity of nodules inoculated with mutant strain was observed, qRT‐PCR was used to determine whether the N‐fixation system, for example nitrogenase genes, is transcriptionally affected in the PrxA‐deficient mutant. The expression of *nifH*, *nifD*, and* fdxN*, three genes involved in nitrogen fixation metabolism and induced in mature bacteroids (Capela, Filipe, Bobik, Batut, & Bruand, 2006), was analyzed in 4‐week‐old nodules by qRT‐PCR (Figure 1b). A significant overexpression of the *katG* gene was also detected in *prxA* mutant bacteroids, suggesting that the nitrogen fixation process induces the transcription of the other antioxidant genes against strong oxidative stress (Figure 1b). In contrast, *nifH*, *nifD*, and *fdxN* were found to be significantly downregulated in HKprxA compared with control nodules. These results confirm the impairment of bacteroid differentiation and N_2_‐fixation function observed in the HKprxA strain.

### 3.6 Quantification of *prxA* gene expression in nodules induced by 7653R

**Figure 2:**
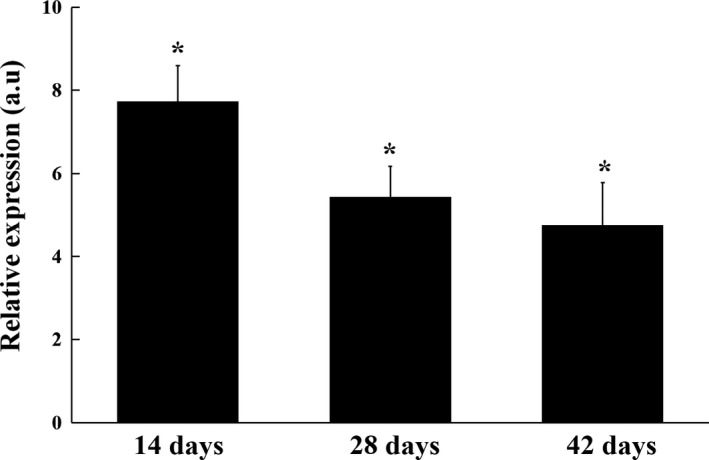
Expression patterns of *prxA* gene in symbiotic nodules. Gene expression levels were examined by real‐time RT‐PCR. Nodules were collected on different days after inoculation with *Mesorhizobium huakuii* 7653R. Relative expression of genes involved in nodule bacteroids compared with 7653R cells growth in AMS Glc. Data are the average from three independent biological samples (each with three technical replicates). Asterisk (*) indicates a significant difference (>2‐fold, *p* ≤ 0.05). AMS, acid minimal salt medium

The relative expression level of the *prxA* gene in root nodules collected at three time points after inoculation was estimated by qRT‐PCR (Figure 2). The expression of the *prxA* gene is upregulated significantly in the early stage (14 d), maturation stage (28 d), and late stage (42 d) of nodule development, and the expression of the *prxA* gene achieved the highest level in nodules at 14 days after inoculation (dai). These results indicated that *prxA* gene expression was induced during symbiosis and was indispensable for bacteroid development and nitrogen fixation.

## 4 DISCUSSION

Reactive oxygen species are involved in the initial stages of the symbiotic interaction between rhizobia and legumes, and low‐nitrogen conditions are required for an optimal symbiotic interaction. Prxs are antioxidant and chaperone‐like proteins critical for maintaining certain levels of ROS. In *Rhizobium*, the expression of the *Rhizobium* sp. NGR234 *prxS* gene, encoding a class of atypical 2‐Cys peroxiredoxins, is highly induced in bacteroids, and an abundant presence of *R. etli* CFN42 PrxS was found in the nodule bacteroid proteome. Although a single *prxS* mutant is not affected in its symbiotic abilities or defence response against oxidative stress, a *prxS*‐*katG* double mutant displays only half of the normal nitrogen fixation capacity (Dombrecht et al., 2010). Here, we focus on a Prx5‐like *prxA* single‐mutant strain of *M. huakuii* that is affected with regard to its nitrogen fixation capacity and oxidative stress response.

Phylogenetic analysis and secondary and tertiary structure predictions indicate that *M. huakuii* PrxA belongs to the peroxiredoxin‐5‐like subfamily, which includes a novel thioredoxin peroxidase mainly identified in mammals (Yuan et al., 2004). A study of human peroxiredoxin 5 family members suggested that they are involved not only in oxidative stress protection mechanisms by reducing apoptosis but also in cell differentiation and signal induction (Yuan et al., 2004). Mutation of *M. huakuii prxA* did not affect the growth of free‐living bacteria but displayed decreased antioxidative capacity under the conditions of the organic oxide CUOOH. In eukaryotes, peroxiredoxins participate in the antioxidant response by catalyzing the reduction of organic hydroperoxides (Abbas, Riquier, & Drapier, 2013). Unlike *Rhizobium* sp. NGR234, in which prxS mutants are not affected by the defence response against oxidative stress under free‐living conditions, a mutation of *M. huakuii prxA* can significantly increase the defence response against H_2_O_2_ at higher concentrations. In *Rhizobium*, defences against peroxide damage include the peroxiredoxins and two catalases namely: *KatG* (catalase HPI) and *KatE* (catalase) (Uhlich, 2009). The PrxA‐deficient *M. huakuii* mutant has increased tolerance of exogenous H_2_O_2_. This cell cytotoxicity was relieved to the wild‐type level by inducing transcription of *katG* and *katE* genes (Figure 1). Glutathione reductase activity and intracellular H_2_O_2_ content were measured in the mutant, showing a significantly high level of glutathione reductase activity and low H_2_O_2_ content compared to the wild‐type when PrxA was not present (Table 3).

**Figure 3:**
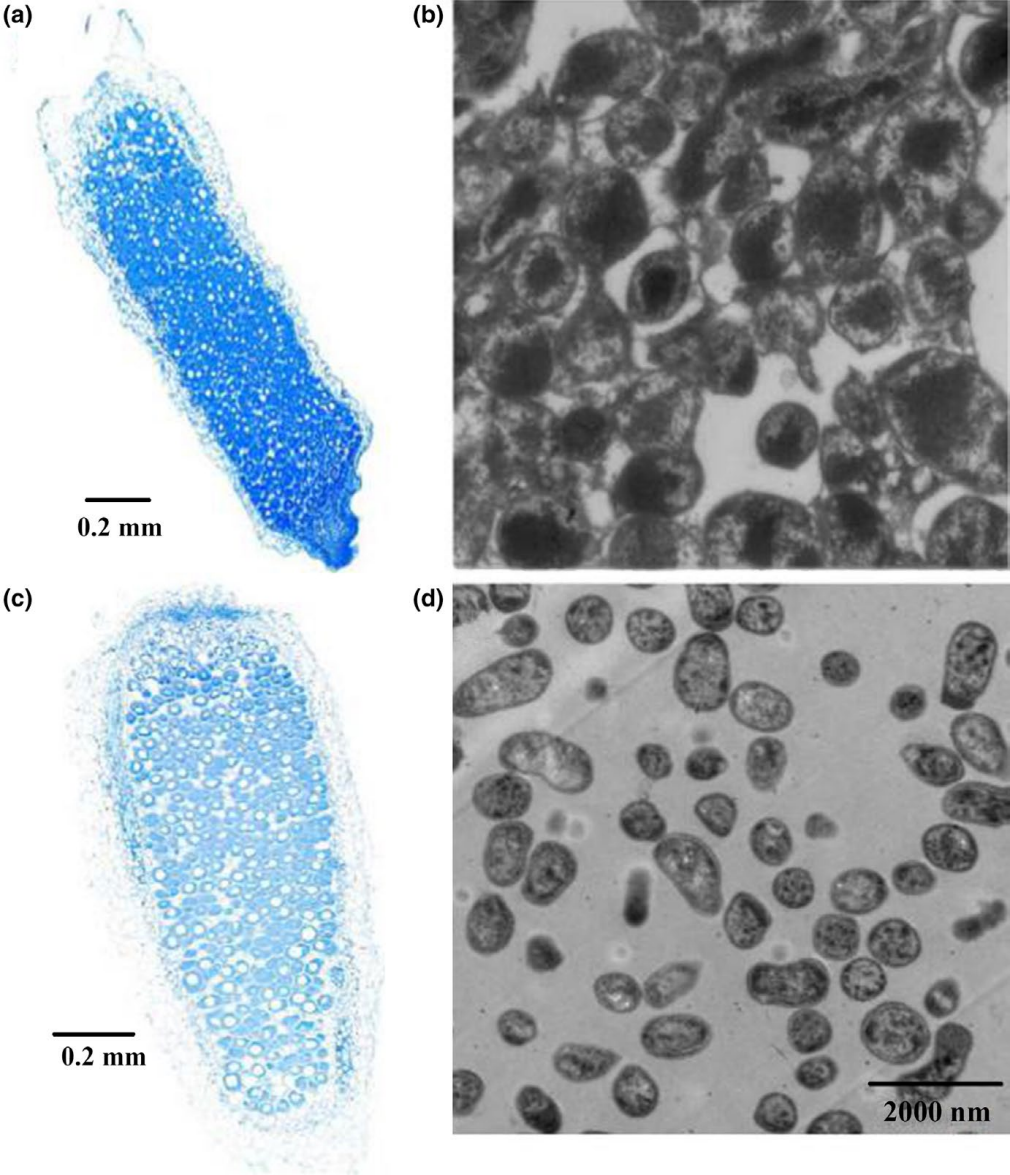
Structure of 4‐week‐old *Astragalus sinicus* nodules and bacteroids. Nodules were induced by *Mesorhizobium huakuii* 7653R (a and b), HKprxA (c and d). Scale bars = 0.2 mm (a and c), 2000 nm (b and d)

The nitrogen fixation phenotype of PrxA‐deficient mutants was assessed, and the *prxA* mutant showed a large reduction in the nitrogen‐fixing activity of root nodules (reduced to approx. 50%), although the total number of nodules/plant (*p* ≤ 0.05) was not affected. In contrast, the symbiotic phenotype of nodules on plants inoculated with *R. etli prxS* mutants was reported to be similar to those inoculated with wild‐type strains, but a *prxS* and *katG* double mutant has significantly reduced (>40%) nitrogen fixation capacity (Yuan et al., 2004). Moreover, a notable feature of our study was the concomitant existence of partially effective nodules. Thus, PrxA is important for both nitrogen fixation efficiency and efficacy of nodule formation. Microscopic analysis of *prxA* mutant nodules showed that they had thickened cell walls in the cortex and a clearly decreased number of bacteroids (Figure 3). Moreover, the size and structure of the bacterial strain in the mutant nodule cells indicated a general lack of bacterial differentiation into bacteroids. The wild‐type 7653R nodule contained the nodule meristem, the infection zone, and the nitrogen‐fixing zone; however, infection with the *prxA* mutant strain resulted in a narrower infection zone (Figure 3a,c). In rhizobia, Kats such as KatA, KatB, KatC, and KatG, are important catalases for defence against peroxide damage. For the *Sinorhizobium meliloti* catalases, the *katA‐katC* and *katB‐katC* double mutants show reduced nodulation (Jamet, Sigaud, Ghislaine, Puppo, & Hérouart, 2003). *Rhizobium etli prxS* mutants are not affected in their symbiotic capacity, whereas a *prxS* and *katG* double mutant displayed reduced symbiotic abilities. In contrast, the *M. huakuii prxA* single mutant showed a large reduction in the nitrogen fixation capacity of nodules. Although *R. etli prxS* and *M. huakuii prxA* are strongly expressed during the symbiotic interaction with plant hosts, these results show a functional redundancy among the catalases during symbiosis. Contrary to the altered nodulation capacity of* R. etli prxS* and *KatG*, *M. huakuii prxA* is essential during symbiosis. The fact that *M. huakuii* KatG is active in the *prxA* mutant bacteroids suggests that PrxA deficiency acts as a functional signal in the nodules for rhizobia. Other *M. huakuii* catalase genes, such as *katG,* are activated to partially restore peroxiredoxin activity. The expression of the nitrogen‐fixation genes *nifH*, *nifD*, and *fdxN* in the *prxA* mutant was significantly lower than in the control nodules. There are several examples indicating that bacterial differentiation is induced by nodule‐specific cysteine‐rich peptides, which resemble the activities of defensins and antimicrobials. Thus, considering the lack of bacterial differentiation in the *prxA* mutant, it is possible that PrxA reacts directly with these peptides to regulate their activity via thioredoxin reductase activity (Benyamina et al., 2012).

## CONFLICT OF INTERESTS

The authors declare no conflict of interest.

## AUTHOR CONTRIBUTIONS

G.C conceived and designed experiments and S.W., T. L., Q. X., and G. C. contributed to the writing of the manuscript. S.W., T. L., Q. X., and K. X. conducted experiments.

## ETHICS STATEMENT

None required.

## Data Availability

All data are provided in full in the results section of this paper apart from *Mesorhizobium huakuii* 7653R genome sequence which is available at https://www.ncbi.nlm.nih.gov/nuccore/NZ_CP006581.
